# Quantitative muscle MRI and functional measures in a cohort of late onset GSD II patients

**DOI:** 10.1186/1471-2474-14-S2-P11

**Published:** 2013-05-29

**Authors:** O Musumeci, M Gaeta, E Barca, A Mileto, G Vita, A Toscano

**Affiliations:** 1 Department of Neurosciences, Psychiatry and Anesthesiology, University of Messina, Messina, Italy; 2 Department of Radiology, University of Messina, Messina, Italy

## Introduction

Adult onset GSDII presents with a wide spectrum of clinical features and variable rates of progression. Three main clinical presentations have been observed: a myopathy with limb girdle muscle involvement (LGMD), asymptomatic hyperCKemia (HCK), and respiratory muscle deficiency preceding limb-muscular weakness. Our study used a new MRI technique with quantitative analysis of muscle fat fraction (MFF) to explore different patterns of muscle involvement and to analyze possible correlations with different clinical aspects in 12 patients with late-onset GSDII.

## Methods

12 patients with late-onset GSDII were recruited before starting enzyme replacement therapy and were divided into three cohorts based on clinical presentation (LGMD, HCK, or respiratory insufficiency). Clinical assessments included the Walton scale, 6MWT, GSGC, FVC% and MRC scores. The muscle MRI protocol was performed using dual-echo, dual-flip-angle spoiled gradient-recalled imaging, which allowed us to accurately quantify and display the muscle fat fraction (MFF). We evaluated the MFF of all respiratory muscles in 12 ambulant patients with late-onset Pompe disease. MRIs included the lower girdle, paraspinal, abdominal, and respiratory muscles. Statistical correlations were performed using the Spearman Rho test.

## Results

MFF of the lower girdle muscles was correlated with functional muscle measures such as the 6MWT, GSGC, and MRC. In particular we found a positive correlation with ileopsoas and gluteus muscles. Moreover, we found a statistically significant correlation between the degree of diaphragm atrophy and pulmonary dysfunction, especially in the upright position.

## Conclusion

MFF is a reliable, relatively fast, and sensitive technique for visualizing muscle involvement, and it provides an accurate fat quantification when compared to muscle biopsy findings in different neuromuscular disorders. In the present study, we show that this technique can be used to evaluate the degree of muscle involvement and is correlated with clinical data currently used as functional outcome measures in Pompe disease. Our data suggested that muscle MRI studies may be a useful tool to evaluate muscle impairment even in the early stages of disease.

